# Prognostic factors affecting outcomes in fistulating perianal Crohn’s disease: a systematic review

**DOI:** 10.1007/s10151-017-1647-3

**Published:** 2017-06-20

**Authors:** G. C. Braithwaite, M. J. Lee, D. Hind, S. R. Brown

**Affiliations:** 1 University of Sheffield Medical School, Sheffield, UK; 2 Department of General Surgery, Northern General Hospital, Herries Road, Sheffield, S5 7AU UK; 3 Clinical Trials Research Unit, University of Sheffield, Sheffield, UK

**Keywords:** Crohn’s disease, Perianal fistulae, Prognosis, Systematic review

## Abstract

**Background:**

One in three patients with Crohn’s disease will develop a perianal fistulae, and one third of these will achieve long-term healing or closure. A barrier to conducting well-designed clinical trials for these patients is a lack of understanding of prognostic factors. This systematic review sets out to identify factors associated with prognosis of perianal Crohn’s fistulae.

**Methods:**

This review was registered on the PROSPERO database (CRD42016050316) and conducted in line with PRISMA guidelines along a predefined protocol. English-language studies assessing baseline factors related to outcomes of fistulae treatment in adult patients were included. Searches were performed on MEDLINE and Embase databases. Screening of abstracts and full texts for eligibility was performed prior to extraction of data into predesigned forms. Bias was assessed using the QUIPS tool.

**Results:**

Searches identified 997 papers. Following removal of duplicates and secondary searches, 923 were screened for inclusion. Forty-seven papers were reviewed at full-text level and 13, 2 of which were randomised trials, were included in the final qualitative review. Two studies reported distribution of Crohn’s disease as a prognostic factor for healing. Two studies found that CARD15 mutations decreased response of fistulae to antibiotics. Complexity of fistulae anatomy was implicated in prognosis by 4 studies.

**Conclusions:**

This systematic review has identified potential prognostic markers, including genetic factors and disease behaviour. We cannot, however, draw robust conclusions from this heterogeneous group of studies; therefore, we recommend that a prospective cohort study of well-characterised patients with Crohn’s perianal fistulae is undertaken.

## Introduction

Crohn’s disease (CD) is an inflammatory condition which can affect any part of the gastrointestinal tract. It is characterised by chronic inflammation all the way through the intestinal wall. Crohn’s disease typically follows one of three behaviour patterns: inflammation only, stricturing, and penetrating [1]. Penetrating disease is typically characterised by formation of a fistulae (an abnormal connection between two epithelial surfaces). This can happen between intestinal loops (enteroenteric), intestine, and skin (enterocutaneous), or the anorectum and buttock skin (perianal). The incidence of perianal fistulae in CD is around 30% [2].

A fistulae is typically managed with sepsis control, through incision and drainage of any abscess, placement of a seton, and immune modulation by drugs such as azathioprine or infliximab (anti-TNF-α therapy) [3, 4]. A number of alternative surgical procedures might also be considered [3]. In serious cases, a stoma might be offered, often as a prelude to proctectomy [4]. This condition can have a significant impact on patients’ quality of life [5–7]. As few as one in three patients will achieve long-term healing of their fistulae [8]. Consequently, health care costs of anal fistulae in CD are high due to drug therapies [9, 10]. It is not surprising that this condition has been identified as a research priority in two recent research priority setting exercises [11, 12].

The aetiology of CD is complex and multifactorial. Recent genomic studies have identified several loci of susceptibility [13–15]. Several of these genes are implicated in aberrant immune responses. Environmental factors such as smoking are thought to play a key part in disease behaviour [16], as in altered intestinal microbiome [17] [18]. These are baseline disease or demographic factors that might be implicated in disease behaviour and prognosis. On top of these systemic mechanisms, localised mucosal damage and aberrant or failed repair mechanisms likely contribute to persistence of fistulae [2, 19].

Randomised controlled trials (RCTs) are the gold standard in clinical research, and these are sorely needed to guide treatment of fistulating perianal CD. To design trials, we need to balance prognostic factors across study arms to limit confounding and produce reliable results [20].

The aim of the present study was to systematically review the literature and identify baseline prognostic factors relevant to the treatment of fistulating perianal CD.

## Materials and methods

This review was registered on the PROSPERO database (CRD42016050316) and conducted in line with Preferred Reporting Items for Systematic Reviews and Meta-Analyses (PRISMA) guidelines using a predefined protocol.

The inclusion criteria were: publication during or after 1980; study size ≥50 patients with rectovaginal or perianal fistulae; fistulae cause by CD; patients aged 16 years or over; fistulae is baseline health state (startpoint [20]) of the study. Exclusion criteria were: CD without fistulae; paper only reports intervention as opposed to demographic or disease status; covariates; paper only includes treatment outcomes as opposed to analysing by demographic or disease status factors. Publications not in English were also excluded due to resource constraints.

**Fig. 1:**
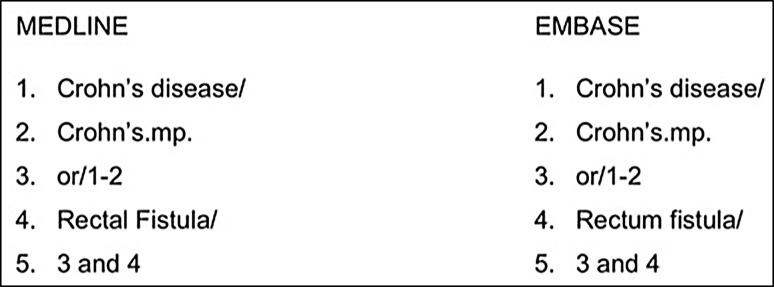
Search terms used in paper selection

Information sources were MEDLINE (1946 to October 26, 2016) and Embase (1974 to October 26, 2016) via Ovid. Searches, which used no limits, combined thesaurus and free-text terms (see Fig. 1).

Results from bibliographic databases were combined with papers through secondary searches of bibliographies and papers of known relevance identified by clinical topic experts, and duplicates removed. Titles and abstracts of citations were screened against the eligibility criteria (by GB), with secondary review and resolution of queries (by ML and DH). Potentially eligible full texts were retrieved and the process repeated, with reasons for rejection recorded.

Data were extracted into predesigned tables (by GB) and findings confirmed (by ML). We extracted data on demographics of the patients and specific details about their condition, including: age; gender; smoking status; duration of disease; location of disease; number of fistulae; treatments; and outcome data on ‘response’ or ‘healing’, that is :fistulae closure, no further discharge from fistulae, or no fistulae recurrence, however defined. Risk of bias (RoB) in individual studies was assessed by two reviewers (GB and ML) using the Quality In Prognosis Studies tool (QUIPS) tool [21]. This tool assesses 6 domains: study participation, study attrition, prognostic factor measurement, outcome measurement, study confounding, and statistical analysis and reporting. We recorded statistical methods used and summary measures, however presented, including odds ratios, relative risks, hazard ratios with confidence intervals, tests of significance (*p* values). We conducted a narrative (descriptive) synthesis with results structured by type of prognostic factor.

## Results

**Fig. 2:**
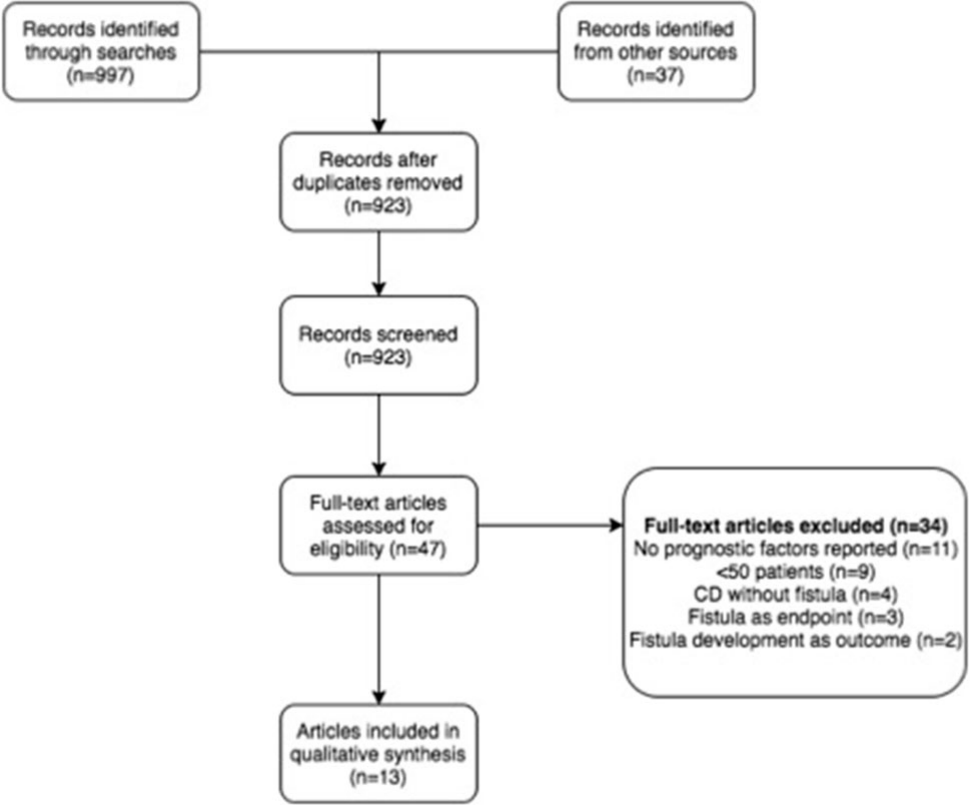
PRISMA flow diagram

The PRISMA study selection flow chart is shown in Fig. 2.

### Study comparisons

Searches identified 997 papers. Following removal of duplicates and secondary searches, 923 were screened for inclusion. Forty-seven papers were reviewed at full-text level. Thirty-four papers were rejected at this stage for the following reasons: no prognostic factors reported (*n* = 11), <50 patients with fistulae caused by CD (*n* = 9), CD without fistulae (*n* = 4), fistulae was an endpoint (*n* = 3), development of fistulae was a factor in natural history of Crohn’s disease (*n* = 2), paper was a narrative review (*n* = 3), or paper was a systematic review (*n* = 2). This left 13 papers for qualitative review.

#### Study demography and design

Of the 13 studies identified, 2 were published between 1995 and the end of 1999 [22, 23], 7 between 2000 and the end of 2009 [24–30], and 4 between 2010 and 2014 [31–34]. Studies and characteristics are summarised in Table 1.

**Table 1:** Summary of included papers and characteristics

Paper	Design	Population	Fistulae (number, anatomy)	Location of disease	Treatment
Bell [24]	Retrospective cohort	Male 41	78 perianal	74 colonic/ileocolonic	Rule based
		Female 46	27 rectovaginal	12 small bowel	Medical (antibiotics, azathioprine, enteral, parenteral feeding)
		Total = 86	169 fistulae 135 complex (80%)	74 colonic/ileocolonic	Simple surgery (drainage, fistulotomy, seton) [most common for simple and complex perianal]
		Age (mean, range) years: 35 (20–91)	‘Simple’—superficial or intersphincteric	12 small bowel	Complex (resection, refunctioning stoma, proctectomy) [most common for rectovaginal]
			‘Complex’—transsphincteric, suprasphincteric, or extraspincteric	74 colonic/ileocolonic	Other (advancement flap, primary repair)
				12 small bowel	
				1 perianal	
				57 rectal involvement	
Dewint [31]	RCT	Male 37	70 perianal (110 localisations) anatomy undefined	N/A	Random
		Female 33			34 allocated to ciprofloxacin (and made it to completion)
		Total = 70			36 allocated to placebo
		Age (mean, range) years: 36.1 (18–70)			Randomisation was performed through a centralised randomisation schedule in a 1:1 ratio
		Smokers 22 (12 in cipro., 10 in placebo)			
Loffler [25]	Prospective cohort	Male 49	45 rectovaginal	Anorectal/rectovaginal 144	Rule based
		Female 98	101 perianal	Colon 141	292 operations on 146 patients
		Total = 147 (1 patient excluded)	Classified according to Parks et al.		38% major surgery, 62% minor surgery
		Age (mean, range) years: 33 (17–68)	Extrasphincteric 34		Minor surgery (lay open-44, fistulae excision-41, fistulae curetting-25, seton drainage-71, fibrin glue application-1)
			Suprasphincteric 24		Major 1 surgery (endorectal advancement flap-34, levatorplasty/sphincteroplasty-20)
			Submucosal 22		Major 2 surgery (ostomy-17, Hartmann’s procedure-10, proctectomy/perianal resection-29)
			Transsphincteric 21		
Luna-Chadid [26]	Prospective cohort	Male 57	59 Perianal	Ileal 33	Rule based
		Female 51	12 Rectovaginal/enterovesical	Colonic 19	All treated with infliximab
		Total = 108		Ileocolonic 55	
		Age (mean) years: 38 Smokers: 54	Fistulae anatomy: Entereocutaneous or Perianal or Internal or Rectovaginal or Enterovesical 194 Localisations		
Present [23]	RCT	Male 44	85 Perianal (anatomy not defined)	Ileum 14	Random
		Female 50		Colon 26	Randomly assigned to infliximab or placebo
		Total = 94		Both ileum and colon 54	
		Age (mean years: 37.2)			
Gaertner [27]	Retrospective cohort	Male 105	226 Perianal, 254 Localisations Classified by method described by Parks et al.	Ileocecal 81	Rule based
		Female 121	‘Complex’—if there are multiple fistulae tracts and extension of tracks above the dentate line	Perianal 62	Underwent operative treatment only (147) or operative treatment and infliximab (79)
		Total = 226	Intersphincteric 103	Colon 51	
		Age (mean, range) years: 39 (16–83) Smokers 32	Transsphincteric 91	Small bowel 20	
			Complex 35	Terminal Ileum 12	
			Extrasphincteric 14		
			Suprasphincteric 11		
Angelberger [59]	Prospective cohort	Male 28	54 Perianal Fistulae anatomy is not defined	Ileocolonic 39	Rule based
		Female 24		Colonic 13	49 treated with ciprofloxacin
		Total = 54			3 treated with metronidazole for 7 weeks
		Age (mean, range) years: 36 (22–61)			
		29 smokers			
Bougen [32]	Retrospective cohort	Male 61	158 perianal 28 simple 128 complex	Ileal 19	Rule based
		Female 95		Colonic 63	IFX administrated
		Total = 156		Ileocolonic 70	
		Age (mean) years: 30		Upper digestive tract 8	Episodic if administrated on relapse of symptoms
					Scheduled if every 8 weeks
Dejaco [29]	Prospective cohort	Male 27	52 Perianal classified according to Parks et al. Superficial 2	Ileocolonic 39	Rule based
		Female 25	Intersphincteric 17	Colonic 13	All treated with ciprofloxacin and/or metronidazole
		Total = 52	Transsphincteric 14		
		Age (mean, range) years: 39 (22–63)	Suprasphincteric 2		
		Smokers 32	Extrasphincteric 1		
			Complex fistulae 7		
			Unclassified 9		
Freire [33]	Prospective cohort	Male 87	203 perianal Classified as ‘simple’—superficial (intersphincteric or low transsphincteric), painless, with a single external opening and no evidence of rectovaginal involvement or anorectal stricture or ‘complex’—fistulae is located high (high transsphincteric, extrasphincteric, or suprasphincteric), may be associated with pain, can potentially involve multiple external openings, and may be associated with rectovaginal fistulae and/or anorectal stricture	N/A	Rule based
		Female 116			Antibiotic treatment; all given metronidazole and 28 also received ciprofloxacin
		Total = 203			
		Age (mean) years: 36.6			
		Male 35.9			
		Female 37.1			
Haennig [34]	Prospective cohort	Male 39	12 Rectovaginal	Perineum 56	Rule based
		Female 42	69 Perianal	Rectum 34	62 had surgery, drainage with a loose seton—all given infliximab for median of 4.9 months
		Total = 81	Simple or complex according to the classification of the American Gastroenterology Association	Ileum 6	
		Age (mean) years: 31; BMI (kg/m^2^) = 20 Smokers 23	71 Complex	Colon 32	
				Ileocolonic 42	
Makowiec [37]	Prospective cohort	Male 37	75 Perianal—14 Complex	Ileal 9	Rule based
		Female 53	15 Anovaginal 50 Transsphincteric (includes 15 anovaginal)	Colitis 31	Standard treatment was a high dose of corticosteroid therapy
		Total = 90	24 Subcutaneous	Ileocolitis 50	36 given prednisolone (6 also received azathioprine)
			4 Intersphincteric		2 received azathioprine alone
			11 Ischiorectal		These 38 were classified as receiving immunosuppressive therapy
			1 Suprasphincteric		9 were given oral metronidazole
					12 received steroids
Michelassi [30]	Prospective cohort	Male 102	51 fistulae in ano	N/A	Rule based
		Female 122	20 Rectovaginal		Surgery for all patients
		Total = 224			Setons used in fistulae
		Age (mean, range) years: 38 (17–82)			

*BMI* body mass index, *N/A* not available

All studies took place in the USA (*n* = 3) [23, 27, 30]) or Europe (Germany (*n* = 3) [22, 25, 28], France (*n* = 2) [32, 34], the UK (*n* = 1) [24], the Netherlands (*n* = 1) [31], Austria (*n* = 1) [29], Spain (*n* = 1) [26], and Portugal (*n* = 1) [33]). The institutional setting was a teaching hospital in all cases.

Ten of the studies were prospective: either observational (*n* = 8) [22, 25, 26, 28–30, 33, 34] or RCTs (*n* = 2) [23, 31]. The remaining 3 studies were retrospective [24, 27, 35]. The follow-up period for studies ranged from 7 weeks to 27.3 years.

Different statistical methods were used to evaluate results. The techniques used were Fisher’s exact test (*n* = 9) [23–25, 27–31, 33], Chi-square test (*n* = 7) [23, 25–27, 30, 31, 33], mean with standard deviation (*n* = 5) [26, 29, 31, 33, 34], Mann–Whitney *U* test (*n* = 4) [24, 28, 31], Kaplan–Meier method (*n* = 4) [22, 25, 32, 34], log-rank test (*n* = 4) [22, 25, 32, 34], Cox proportional hazards regression model (*n* = 3) [22, 32, 34], 95% confidence Intervals (*n* = 2) [26, 33], odds ratios (*n* = 2) [23, 33], Wilcoxon rank tests (*n* = 2) [22, 28], median with interquartile range (*n* = 2) [31, 32], log-likelihood ratio (*n* = 1) [26], Kruskal–Wallis test (*n* = 1) [25], Kolmogorov–Smirnov test (*n* = 1) [33], and Hardy–Weinberg test (*n* = 1) [33]. Statistical methods and potentially confounding variables recorded are shown in Table 2.

**Table 2:** Additional baseline demographics and statistical tests used to assess prognostic factors

Paper	Previous treatment	Other perianal manifestations or stoma	Time period	Follow-up (mean, range)	Duration of CD	Statistical methods
Bell [24]	N/A	N/A	Jan 1993–Dec 1994	5.5 years (7 weeks–27.3 years)	8 years (0–32 years)	Mann–Whitney *U* test (nonparametric comparisons). Fisher’s exact test for associations between data sets.
Dewint [31]	Concomitant use of thiopurine derivates, methotrexate, 5-aminosalicylic, oral corticosteroids	11 had previous stoma	Sept 2008–March 2011	24 weeks	N/A	Distributions between treatment groups were compared by X2 or the Fisher exact test. Continuous variables were summarised by using median and IQR or mean and SD, and their distributions between treatments were compared with Mann–Whitney test. Frequencies of response were compared between treatments using X2 or Fisher’s exact test
Loffler [25]	27 on immunosuppressants at time of trial	N/A	1991–2001	48 months	N/A	Using SAS software, difference in no. of operations between fistulae type was calculated by Kruskal–Wallis test. Number of protectomies according to fistulae type with Fisher’s exact test
Luna-Chadid [26]	Azathioprine 73	N/A	Oct 1999–March 2001	At least 4 weeks	9 years	Comparisons between independent proportions were carried out by Chi-square test
	Corticosteroids 59					
	5-Aminosalicylates 81					
	Metronidazole 72					
	Ciprofloxacin 35					
Present [23]	Corticosteroids 33	Previous stoma excluded	N/A	N/A	12.4 years	The primary analysis was performed with the intention-to-treat principle and included all patients who were assigned to treatment. 1. The Mantel–Haenszel Chi-square test for a linear dose response in the proportion of patients in whom the primary endpoint occurred. 2. If significant, Fisher’s exact test was used to compare the proportion of patients achieving the primary endpoint in each of the two infliximab groups with the placebo group. Odds ratios were used to assess the consistency of benefit of infliximab treatment. Analysis of the proportion of patients with complete response was performed with the same methods for analysis of the primary endpoint. Continuous variables were compared by analysis of variance of the van der Waerden normal scores
	Mercaptopurine or azathioprine 38					
	Aminosalicylates 52					
	Antibiotics 28					
Gaertner [27]	84 had previous surgery	N/A	March 1991–Dec 2005	30 months (6–216)	7 years (0.08–38)	Pearson Chi-squared and Fisher’s exact tests were performed to compare baseline patient characteristics and differences in healing between treatment groups. Fisher’s exact test was performed to compare differences in healing between patients based on type of fistulae, initial site of CD, and operative treatment. *p* < 0.05 was considered significant. All calculations were performed by using the GraphPad InStat 3 statistics programme
Angelberger [59]	31 previous surgery 5-aminosalicylic acid, sulphasalazine—21	N/A	N/A	N/A	3.9 years (0.1–26.4)	Fisher exact test for 2 × 2 frequency tables. Comparison of the HBD-2 gene copy number and number of draining fistulae between the patient groups was performed by the Wilcoxon signed rank test and Mann–Whitney *U* test, respectively. All calculations were done by SAS and SPSS statistical software
	Steroids—11					
	Immunosuppressants-23					
Bougen [32]	Major abdominal surgery 44 Purine analog 51	N/A	Jan 1998–Sept 2011	5 years	3.8 years (0–30)	Quantitative variables were described as median and percentile (IQR) Categorical variables were presented as counts and per cent of cohort. Four events were defined: events were analysed using survival analysis. Cumulative probabilities of fistulae closure, recurrence of PCD, or abscess were estimated using Kaplan–Meier method. To identify predictive factors, we performed a univariate analysis using the log-rank test. When considering the continuous variables for dichotomous analysis, cut-off values were determined using receiver operating characteristic analysis to reduce the risk of bias related to arbitrarily defined cut-off and identify the optimal cut-off using each outcome as a classification variable. To identify independent predictors of surgery using a multivariate analysis, all significant variables in the log-rank test were retained in the model and integrated into a Cox proportional hazards regression model
	Methotrexate 6					
	Adalimumab 3					
	Concomitant:					
	Steroids—45					
	Purine analog—82					
	Methotrexate—8					
	Antibiotics—90					
Dejaco [29]	Perianal surgery 32 Concomitant: Aminosalicylates	Previous stoma excluded	July 1999 –Feb 2002	28.1 months	11 years (2–35)	Results are expressed as the mean ± standard deviation. Comparison of PDAI scores, leucocyte counts, and C-reactive protein levels before and during treatment was analysed by the paired exact Wilcoxon signed rank test. For the detection of differences between response rates in patients receiving different types of medication, Fisher’s exact test was used. Multivariate logistic regression analysis was performed by SAS in order to assess the simultaneous effects of smoking, azathioprine administration, and duration of fistulising disease on treatment response at week 20
Freire [33]	Concomitant	N/A	N/A	N/A	N/A	Categorical variables were expressed as frequency and percentage, and corresponding contingency tables were analysed with Pearson’s Chi-square test or Fisher’s exact test, OR were determined with 95% CI. Continuous variables were summarised using mean ± standard deviation. These variables were tested for normal distributions using the Kolmogorov–Smirnov test. The Student’s t test was employed to compare means of continuous variables and normally distributed data; otherwise, the Mann–Whitney U test was applied. All variants studied were in Hardy–Weinberg equilibrium. Data were analysed using the Statistical Package for Social Sciences
	5-Aminosalicylic acid = 34					
	Steroids = 6					
	Azathioprine (<3 months) = 9					
	Azathioprine (≥3 months) = 7					
Haennig [34]	N/A	N/A	2000–2010	63.8 months (2–263)	N/A	Quantitative variables are given as mean ± SD and median with range. The time to complete closure and its relation to the duration of seton drainage or infliximab treatment was determined using the Kaplan–Meier method, and significance was demonstrated using the log-rank test. Cox uni- and multivariate analysis was used to determine the effect of clinical variables on closure. Factors significantly associated with closure in univariate analysis were applied to a restricted multivariate mode
Makowiec [37]	Previous surgery: 41 for intestinal disease	80 had abscesses	May 1989 –Oct 1992	22 months (6–44) Follow-up ended Dec 1993	8 years (0–22)	Inactivation of perianal fistulae and abscesses, healing, reopening, and symptomatic recurrence rates were analysed using Kaplan–Meier survival estimates. Patients were considered at risk until the event occurred (inactivation, healing, recurrence) or until the last follow-up examination. Factors influencing healing or symptomatic recurrence were analysed by log-rank and Wilcoxon rank tests (univariate analysis). The data underwent further independent analysis using multiple regression according to the proportional hazard model (Cox regression analysis)
	69 for perianal fistulae or abscesses	7 stoma				
Michelassi [30]	N/A	Perianal abscesses 36 Anal stenosis 40 Incontinence 11	Oct 1984–May 1999	N/A	N/A	All data were transcribed on a relational database software programme for subset query extraction and analysis. Where appropriate, nominal variables were compared by using Chi-square analysis or single-tailed Fisher exact test. Statistical calculations were made with the aid of a statistical software package (Minitab 10.1 for Windows; Minitab, Inc, State College, PA, USA
		Stoma 5				

*CD* Crohn’s disease, *N/A* not available, *PDAI* perianal disease activity index

#### Outcomes

Identified prognostic factors were related to various outcome measures defined differently in the 13 papers. Common outcome terms were healing, response, complete response, partial response, and recurrence. A summary of various definitions and common ‘headings’ used is presented in Table 3.

**Table 3:** Common outcome groups and definitions used

Common outcome measure	Definition given in paper
‘Healed’/‘healing’/‘complication healed’ (*n* = 4)	No discharge on history or examination, with healing of the external opening [24]
	Complete closure of fistulae without sign of activity or pain for at least a month [37]
	Complete healing or successful dilation of anal stenosis, after surgical intervention [30]
	Non-defined [27]
Response (*n* = 3)	≥50% reduction in fistulas [31]
	Maintained fistulae healing; PDAI 2.8 ± 2.4 [29]
	Absence of fistulae drainage, even after compression for at least 4 weeks [33]
Complete response (*n* = 4)	The complete cessation of drainage from all fistulas despite gentle finger compression [26]
	Absence of any draining fistulas [23]
	Absence of any drainage fistulas despite gentle finger compression [28]
	PDAI 0.8 ± 1.0 fistulae closure or absence of any draining fistulas despite gentle finger compression [29]
Partial response (*n* = 2)	At least 50% reduction from baseline in the number of fistulas or drainage for at least 4 consecutive weeks after the discontinuation of drug infusions [26]
	Reduction of 50% or more from baseline in the number of draining fistulas [28]
Recurrence (*n* = 4)	Presence of fistulae openings among patient who experienced fistulae closure [32]
	Reopening of a former track or presence of new fistulae after primary response [34]
	Reappearance of active perianal fistulas or associated abscesses after prior inactivation or healing [37]
	Recurrence of the same or different complication after a period of complete healing [30]

*PDAI* perianal disease activity index

#### Bias

Risk of bias findings are presented in Table 3. Overall risk of bias in the studies was judged to be low for 7 [26, 28, 29, 31–34, 36] and moderate for 6 studies [23–25, 30, 37] [24]. Study attrition was typically low. The domains most commonly at high risk of bias were study confounding (*n* = 5) [22, 24, 25, 28, 30] and statistical analysis and reporting (*n* = 6) [26, 30–33, 37]. This bias assessment is shown in Table 4.

**Table 4:** Risk of bias using QUIPS tool

	Overall risk of bias	1. Study participation	2. Study attrition	3. Prognostic factor measurement	4. Outcome measurement	5. Study confounding	6. Statistical analysis and reporting
Bell [24]	Moderate	L	L	L	M	H	M
Dewint [31]	Low	L	L	M	L	M	H
Loffler [25]	Moderate	M	L	M	M	H	M
Luna-Chadid [26]	Low	L	L	H	L	L	H
Present [23]	Moderate	M	M	L	L	M	M
Gaertner [39]	Moderate	L	L	H	H	M	M
Angelberger [61]	Low	L	L	M	L	H	M
Bougen [32]	Low	L	L	L	M	L	H
Dejaco [29]	Low	L	M	M	L	L	M
Freire [33]	Low	L	L	L	M	L	H
Haennig [34]	Low	M	M	L	L	L	M
Makowiec [37]	Moderate	L	M	M	L	H	H
Michelassi [30]	Moderate	M	L	M	L	H	H

*L* low risk of bias, *M* moderate risk of bias, *H* high risk of bias

*QUIPS* Quality in Prognostic Studies

#### Prognostic factors

Prognostic factors were divided into those associated with patient characteristics, disease characteristics, and environmental characteristics. These are summarised in Table 5.

**Table 5:** Studies and prognostic factors assessed

Paper	Clinical endpoints	Significant prognostic factors	Insignificant prognostic factors
Bell [24]	‘Healed’—no discharge on history or examination, with healing of the external opening	Rectal Crohn’s made proctectomy more likely than those with no rectal involvement (*p* = <0.001)	Complex did not take significantly longer to heal than simple (*p* = 0.69)
	‘Persistent fistulae’—not defined	Complex perianal took an average of 6 procedures over 2 or more years	The presence of a rectovaginal fistulae was not predictive of the need for a proctectomy (*p* = 0.25)
	‘Maintenance with a seton’—not defined	This is significantly more procedures than simple (3 treatments, *p* = 0.002)	No association between presence of rectal CD and rectovaginal fistulae (*p* = 0.085)
	‘Sepsis’—if an abscess formed at the fistulae site	This is significantly more than rectovaginal (3 treatments, *p* = 0.01)	
	‘None healed’ ‘death’	This is significantly more procedures than abdominal wall (2 treatments, *p* = 0.0005)	
		This is significantly more time than internal fistulae (1 treatment, *p* = 0.002)	
		Complex fistulae took on average 42.8 months to heal	
		Rectovaginal fistulae took significantly shorter time to heal (median of 26 months) than perianal fistulae (*p* = 0.05)	
		Abdominal wall fistulae took significantly shorter time to heal (median of 6.3 months) than perianal fistulae (*p* = 0.0001)	
		Enteroenteric took significantly shorter time to heal (median of 9.4 months) than perianal fistulae (*p* = 0.03)	
Dewint [31]	‘Response’ –	None	Sex (*p* = 0.74)
	≥50% reduction in no. of fistulae		Race, Caucasian versus other (*p* = 0.39)
	‘Remission’ –		Seton (*p* = 0.90)
	100% closure of draining fistulae		Stoma (*p* = 0.30)
			Smoker (*p* = 0.64)
			Previous treatment with infliximab (*p* = 0.63)
Loffler [25]	‘Long-term success’—whether or not patients have fistulae persistence or recurrence over 60 months	98% of patients with anorectal or rectovaginal disease also had a manifestation in colon/rectum. This was significantly higher than in patients without anorectal or rectovaginal fistulae (*p* < 0.001)	Complex fistulae in comparison with simple fistulas, there was a strong trend to a difference in outcome of 5 years (*p* = 0.2113)
Luna-Chadid [26]	‘Complete response’—the complete cessation of drainage from all fistulas despite gentle finger compression	None	Age
	‘Partial response’—at least 50% reduction from baseline in the number of fistulas or drainage for at least 4 consecutive weeks after the discontinuation of drug infusions		Sex
	‘Response for rectovaginal fistulae’—closure documented by physical examination		Smokers
			Duration of fistulising disease
			(no *p* value given, just says the *p* value is not significant)
Present [23]	‘Complete response’—absence of any draining fistulae	Males (*p* < 0.001)are more likely than females (*p* = 0.28) to reach primary endpoint when in infliximab group as compared to placebo group	None
	A fistulae was considered to be closed when it no longer drained despite gentle finger compression		
Gaertner [27]	‘Healing’—not defined	None	There were no significant associations found between fistulae healing and the duration of CD, initial site of CD, previous fistulae disease, and cigarette smoking
Angelberger [59]	‘Complete response’ -absence of any draining fistulae despite gentle finger compression	Complete fistulae response was significantly higher in patients with NOD2/CARD15 wild type	Median HBD-2 gene copy number was not significantly different between the responders and non-responders (*p* = 0.92)
	‘Partial response’—reduction of 50% or more from baseline in the number of draining fistulae	(*p* = 0.02)	Duration of perianal fistulating disease (*p* = 0.844)
			Smoking (*p* = 0.239)
			Association between complete response and median number of draining fistulae (*p* = 0.18)
			Rate of patients with more than one draining fistulae (*p* = 0.32)
Bougen [32]	(1) Fistulae closure = absence of any draining by fistulae openings at one visit	Significant predictors of perianal fistulae closure: prior abdominal surgery	Sex (*p* = 0.12) HR 1.46 (95% 0.89–2.35)
	(2) Recurrence of PCD = presence of fistulae openings among patient who experienced fistulae closure	(*p* = 0.0097) HR 0.43 (95% CI 0.21–0.8)	
	(3) Recurrence of abscess after IFX initiation		
	(4) Sustained fistulae closure for patients without any recurrence		
Dejaco [29]	‘Response’—maintained fistulae healing, PDAI 2.8 ± 2.4	The duration of fistulising disease was a significant prognostic factor (*p* = 0.04)	Smoking (*p* = 0.3)
	‘Complete Response’—PDAI 0.8 ± 1.0, fistulae closure or absence of any draining fistulae\despite gentle finger compression		
	‘No response’ –		
	PDAI 7.4 ± 3.1		
Freire [33]	‘Response’—absence of fistulae drainage, even after compression for at least 4 weeks	Clinical response of perianal fistulae to antibiotics was significantly higher in patients without the CARD15 mutation (*p* = 0.041)	None
		OR 8.16 (95% CI 0.97–68.74)	
Haennig [34]	‘Clinical response’—complete closure of the fistulae track with no further discharge from the opening(s) on the gentle application of pressure	The time for closure of fistulae was significantly shorter for men than women (*p* = 0.03) HR 0.59 (95% CI 0.36–0.96)	Recurrence after initial fistulae closure—tobacco (*p* = 0.41)
	‘Primary response’—closure had been sustained for at least 4 months	11.7 versus 21.0 months	Ileocolonic location of CD (*p* = 0.10)
	‘Recurrence’—reopening of a former track or presence of new fistulae after primary response	The time for closure was significantly shorted for simple fistulae compared to complex fistulae (*p* < 0.001) HR 0.31 (0.16–0.62)	Rectovaginal fistulae (*p* = 0.24)
		2 versus 15.3 months	
		Rectovaginal fistulae took a significantly longer time to close than perianal (*p* = 0.02) HR 0.44 (0.22–0.91)	
		12 versus 30.6 months	
Makowiec [37]	‘Inactivation of perianal fistulas and abscesses’—cessation of purulent discharge from fistulae and disappearance of perianal pain	Ischiorectal and transsphincteric fistulae recurred more frequently than low fistulas (*p* = 0.007)	None
	‘Healing’—complete closure of fistulae without sign of activity or pain for at least a month	Low fistulas had a better prognosis (higher healing rate) than transsphincteric	
	‘Reopening of fistulae’—reappearance of perianal fistulas after prior healing	or ischiorectal fistulas	
	‘Symptomatic recurrence’—reappearance of active perianal fistulae or associated abscesses after prior inactivation or healing	(*p* = 0.015)	
		The presence of rectal disease indicated that a patient was significantly more likely to have recurrence (*p* = 0.041)	
		Fistulae healed better in patients without than in those with rectal disease (*p* = 0.017)	
		If presence of stoma are more likely to heal (*p* = 0.005)	
Michelassi [30]	‘Persistence’—persistence of a complication after surgical intervention	A patient is significantly less likely to heal from a perianal complication when there is rectal involvement (*p* < 0.05)	None
	‘Development’—development of a complication different from the original one as a consequence of surgical intervention	49.1 versus 19.3%	
	‘Recurrence’—recurrence of the same or different complication after a period of complete healing	A patient is significantly more likely to heal when they have a single complication compared to having multiple complications (*p* < 0.05)	
	‘Complication healed’—complete healing or successful dilation of anal stenosis, after surgical intervention	48.6 versus 28.2%	
	‘Sepsis controlled’—anorectal sepsis controlled as consequence of surgery	Patients with rectal involvement had a significantly higher chance of proctectomy (*p* < 0.0001)	
		77.6 versus 13.6%	
		Patients with multiple complications had significantly higher chance of proctectomy (*p* < 0.05)	
		23 versus 10%	

*CD* Crohn’s disease, *PDA* perianal disease activity index, *PCD* perianal Crohn’s disease

### Patient characteristics

Two papers found that patient sex was significant. A RCT of infliximab versus placebo (*n* = 94) found that males were significant more likely than females to reach the primary endpoint (*p* < 0.001) versus (*p* = 0.28) [23]. Another paper (*n* = 81) found that time for closure of fistulae was significantly shorter for men than women, at 11.7 months versus 21.0 months (*p* = 0.03) [HR 0.59, (95% CI 0.36–0.96)] [34]. Three papers found sex had no significant association with outcome. One trial (*n* = 70) found sex was not significant to the ‘response’ of patients (*p* = 0.74) [31] and another (*n* = 108) found no difference between the sexes (*p* > 0.05) [26]. A retrospective study (*n* = 156) found that sex was not a significant prognostic factor. (*p* = 0.12) [HR 1.46, (95% CI 0.89–2.35)] [32]

Only 1 trial (*n* = 108) assessed age as a prospective factor and did not find it to be significant (*p* > 0.05) [26].

Race was evaluated in 1 study (*n* = 70) as ‘Caucasian versus other’ and was found not to be a significant predictor of healing (*p* = 0.39) [31].

Studies did not clearly report baseline/historic use of medications; this was reported as previous or current use of immunosuppression and therefore not included in this study.

### Genetics

Two papers evaluated the clinical response of NOD2/CARD15 variant carriers versus wild-type patients to antibiotic therapy. One study (*n* = 54) found that that complete fistulae response was more likely with wild-type (33 vs. 0%, *p* = 0.02) [28]. The other (*n* = 203) found that those without the mutation were more likely to show clinical improvement when treated with antibiotics (7.7 vs. 40.5%, *p* = 0.041) [33]. Both of these studies relied on fistulae drainage and had small numbers in the variant carrier group; therefore, caution should be exercised in interpreting these results.

### Disease duration and location

A prospective observational study (*n* = 52) found the duration of fistulating disease was a significant prognostic factor, although strength and direction of association was not clearly reported (*p* = 0.04) [29]. Two prospective studies found the duration of perianal fistulating disease was not significant—again measures used to assess this were not clear [26, 28]. A retrospective study (*n* = 226) found no significant associations between fistulae healing and the duration of CD [27].

Two papers reported patients with ileal CD only (in association with perianal disease) were significantly more likely to have better outcomes than those with other disease distributions. One RCT (*n* = 94) noted complete fistulae response was more likely in those with ileal and colonic disease (OR 5.1, *p* = 0.01) than those with isolated colonic disease (OR 2.3 *p* = 0.35) [23]. A retrospective study (*n* = 156) found patients with ileocolonic disease were more likely to achieve fistulae closure [HR 1.59 (1.08–2.34) *p* = 0.017] compared to those with colonic disease [HR 0.86 (0.58–1.27) *p* = 0.54] on univariate analysis [32]. On multivariate analysis, ileocolonic behaviour was positively associated with fistulae healing [HR 1.88 (1.08–3.32) *p* = 0.025]. This finding was not upheld by 1 prospective study (*n* = 81), and 1 retrospective study (*n* = 226) which found no association between fistulae healing and the initial site of CD [27, 34]. Three prospective studies found rectal involvement in CD was a predictor of poor fistulae healing [24, 25, 30].

### Fistulae anatomy

Three papers identified complexity of fistulae anatomy as a prognostic factor. Prospective studies found that compared to simple fistulae, complex fistulae required more treatments (*n* = 86) (*p* = 0.02) [36] and took longer to heal (15.3 vs. 2 months) (*n* = 81) (*p* < 0.001) [HR 0.31 (95% CI 0.16–0.62)] [34]. A retrospective study (*n* = 156) demonstrated that simple fistulae was associated with fistulae closure [HR 2.53 (95% CI 1.43–4.45) (*p* = 0.006)] [32] Another study (*n* = 147) found a trend towards worse outcomes at 5 years for complex versus simple fistulae (*p* = 0.2113) [25].

One study (*n* = 224) found that a patient with multiple fistulae was less likely to achieve healing than a patient with a single fistulae [48.6 vs. 28.2% (*p* < 0.05)] [30]. This was not consistent across all studies [24, 25].

Presence of a rectovaginal fistulae was not thought to be a prognostic factor for overall perianal fistulae healing (*n* = 81) [27].

### Environmental characteristics

Six studies evaluated smoking, and none of these found it to be a significant prognostic factor [26–29, 31, 34]. This is summarised in Table 6.

**Table 6:** Studies assessing smoking as a prognostic factor in outcome of perianal Crohn’s fistulae

Study	Total patients (*n*)	Smokers (*n*)	*p* value	Prospective/retrospective
Dewint [31]	70	22	0.64	Prospective
Luna-Chadid [26]	108	54	>0.05	Prospective
Angelberger [28]	54	29	0.239	Prospective
Dejaco [29]	52	32	0.3	Prospective
Haennig [34]	81	23	0.41	Prospective
Gaertner [27]	226	32	>0.05	Retrospective

## Discussion

To our knowledge, this is the first systematic review to assess prognostic factors in fistulating perianal CD. It has identified candidate prognostic factors including NOD2/CARD15, duration of fistulating disease, distribution of CD, and fistulae anatomy. These require further robust assessment before they can be used to inform research or clinical practice. The challenges to prognostic research in this field are many, including lack of standardised outcome measures and timing of outcome measurement.

The NOD2 and CARD15 variant genes had a significant association with fistulae response to antibiotics in 2 studies [28, 33]. Prior work has found associations between disease severity and expression of the various alleles, particularly with aggressive luminal disease requiring early and repeated surgery [38–40]. This suggests that these are plausible factors related to the prognosis of fistulating perianal CD, although there is insufficient evidence presented at this point to understand strength of association, or modulating factors.

Duration of fistulating disease was significant in 1 study (with unclear direction), but not in 2 others. Long-standing fistulae have been shown to undergo epithelialisation and behave in a similar fashion to skin, and this may reduce the ability to heal [41–43]. If track epithelialisation is the underlying mechanism, then it may be reasonable to consider fistulae duration as a prognostic factor (or a proxy of a prognostic factor).

Disease distribution is possibly a prognostic factor, with ileal disease associated with a better prognosis and colonic or rectal disease associated with a worse prognosis. Guidelines advocate early assessment for proctitis in Crohn’s fistulae, as this impacts clinical strategy and outcome [4, 44, 45]. Proctitis has been associated with higher rates of proctectomy in previous studies, suggesting that this factor has a role in predicting outcomes in these patients [46].

The behaviour of the fistulating process is most likely a factor in healing, both in terms of complexity and number. Those with complex anatomy (multiple branching tracks crossing large proportions of the anal sphincter) are at risk of recurrent sepsis [47]. Unfortunately, terminology used to define ‘complex’ and ‘simple’ is not standard across the literature. Complexity of fistulae anatomy is more than location and number of branches. Magnetic resonance imaging offers the ability to assess volume and length of fistulae tracks [48]. It is plausible that a longer or large-volume fistulae track could take longer to heal than a short- or low-volume track. This is potentially an important prognostic marker and therefore would merit further assessment.

Patient demographics including sex may not have a role to play; the majority of studies reviewed found no relationship between sex and outcome, and those that did identify statistical differences obtained conflicting results. This may reflect sampling issues.

None of the studies reviewed found that smoking was a significant prognostic factor in fistulae outcomes. Smoking has been shown to be associated with poor disease control, and smoking cessation is widely advised in CD [49–51]. Given this, it is interesting that it is not a significant factor here. This could be for a number of reasons: bias of design of studies through definition of smoking (patient reported vs. carbon monoxide testing), or size or sampling of patients; that there is no mechanistic role for smoking in the formation of perianal fistulae; or that disease is already ‘bad’ and smoking has no additive effect.

The number of prognostic factors identified was limited by the number of studies reporting baseline factors with appropriate analysis. Even if cohorts had been well described, it would not have been possible to perform a meta-analysis in this setting as there was little consistency across study endpoints. There were 5 major groups of outcome (healed, response, complete response, partial response, recurrence), with an average of 4 definitions for each outcome. Definition of recurrence was fairly consistent across studies. The definition of healed included an asymptomatic fistulae, a non-draining fistulae on compression, and a change in the perianal disease activity index (PDAI). These are relatively subjective measures; even the PDAI has subjective elements [52], at a single time point. It is clear that there are issues to be addressed before further studies are undertaken to investigate this further.

There are limitations to consider in this review. Initial screening by a single reviewer to select studies and extract data increased the possibility that relevant reports were discarded [53, 54]. Despite this, we had multiple checks in place to support the single reviewer process, including screening of discarded abstracts for key papers by a second reviewer. This, coupled with support from clinical topic experts and a robust bibliography search, meant that we were confident that we had identified the majority of papers reporting prognostic factors.

This study used a broad search strategy to identify as many candidate papers as possible and used a tool appropriate for the assessment of prognostic factors (QUIPS). The validity of the findings is supported by the prognostic role of some reported factors in other aspects of inflammatory bowel disease. There are diminishing marginal returns from the use of databases additional to MEDLINE and Embase, with some such as CINAHL rarely retrieving unique references for many topic areas [55, 56]. For this reason, we believe our search strategy is associated with a low risk of bias.

It is important that any future prognostic study captures the above factors and uses a standardised well-defined outcome measure. A well-conducted cohort study will allow all the above factors to be properly assessed using appropriate multivariate statistical models [57, 58]. Given the prevalence and incidence of perianal CD, it might be possible to use the resulting data to inform novel study designs. Clear understanding of confounding factors might allow for trials within cohorts, Bayesian modelling or interrupted time series as alternatives to classical trial designs.

## Conclusions

This systematic review has identified potential prognostic markers for outcomes in fistulating perianal CD, including genetic factors and disease behaviour. We cannot, however, draw robust conclusions from this heterogeneous group of studies. We recommend that future studies include well-characterised cohorts and use a consistent endpoint for reporting.
